# An analysis of characteristics of post-authorisation studies registered on the ENCePP EU PAS Register

**DOI:** 10.12688/f1000research.12198.2

**Published:** 2017-11-10

**Authors:** Robert Carroll, Sreeram V. Ramagopalan, Javier Cid-Ruzafa, Dimitra Lambrelli, Laura McDonald

**Affiliations:** 1 Real-World Evidence, Evidera, London, W6 8DL, UK; 2 Centre for Observational Research and Data Sciences, Bristol-Myers Squibb, Uxbridge, UB8 1DH, UK

**Keywords:** drug safety, pharmacovigilance, post-authorisation studies, study design

## Abstract

**Background**: The objective of this study was to investigate the study design characteristics of Post-Authorisation Studies (PAS) requested by the European Medicines Agency which were recorded on the European Union (EU) PAS Register held by the European Network of Centres for Pharmacoepidemiology and Pharmacovigilance (ENCePP).

**Methods**: We undertook a cross-sectional descriptive analysis of all studies registered on the EU PAS Register as of 18 ^th ^October 2016.

**Results**: We identified a total of 314 studies on the EU PAS Register, including 81 (26%) finalised, 160 (51%) ongoing and 73 (23%) planned. Of those studies identified, 205 (65%) included risk assessment in their scope, 133 (42%) included drug utilisation and 94 (30%) included effectiveness evaluation. Just over half of the studies (175; 56%) used primary data capture, 135 (43%) used secondary data and 4 (1%) used a hybrid design combining both approaches. Risk assessment and effectiveness studies were more likely to use primary data capture (60% and 85% respectively as compared to 39% and 14% respectively for secondary). The converse was true for drug utilisation studies where 59% were secondary vs. 39% for primary. For type 2 diabetes mellitus, database studies were more commonly used (80% vs 3% chart review, 3% hybrid and 13% primary data capture study designs) whereas for studies in oncology, primary data capture were more likely to be used (85% vs 4% chart review, and 11% database study designs).

**Conclusions**: Results of this analysis show that PAS design varies according to study objectives and therapeutic area.

## Introduction

Randomised trials are considered the gold standard in evaluating the efficacy of new healthcare interventions. However, despite their ability to potentially provide causal estimates of the efficacy of new treatments, their generalisability to patients in the real world is often unclear. Strict criteria excluding patients with comorbidities or those above a certain age may lead to participants in trials differing from the general clinical population ^1, 2^. Observational studies offer a means of further characterising the safety and effectiveness of new healthcare interventions in real world clinical settings ^3^.

Post-authorisation studies (PAS), also referred to as ‘postmarketing surveillance studies’ or ‘phase IV trials’, are an example of this type of research which aims to demonstrate the utilisation and safety profile of drugs following their regulatory approval. While these studies are predominantly non-interventional, PAS can include interventional study designs (as per the definition of post-authorisation safety study in GVP VIII). Historically, observational PAS have been criticized to be of poor quality and open to bias in order to increase sales at the cost of scientific rigor ^4^. European pharmacovigilance legislation was introduced in an effort to increase transparency and ensure such studies are methodologically robust ^5^.

Since July 2012 all PAS that are imposed as a condition of granting marketing authorisation are required to be published in a publicly available register. In Europe, the main register for PAS is the EU PAS Register and is held by the European Network of Centres for Pharmacoepidemiology and Pharmacovigilance (ENCePP). While one previous study has examined broad characteristics of studies registered on the EU PAS, the current study aimed to build on this work by further characterising studies requested by the EMA to understand trends in study design, data sources utilised, and the relationship to therapeutic area. As PAS are becoming increasingly mandated by the European Medicines Agency (EMA) and compliance is high ^6^, approved PAS protocols can likely provide insights into the successful design of future studies.

## Methods

Analysis focused on all PAS requested by the EMA which were recorded on the EU PAS Register as of 18 ^th^ October 2016. The EU PAS Register is accessible via the ENCePP website which is hosted and maintained by the European Medicines Agency (EMA). All studies in category 1 (imposed as a condition of marketing authorisation), category 2 (obligation of marketing authorisation), and category 3 (required by the risk management plans) on the EU PAS register were included. These studies currently (as at 6 ^th^ November 2017) represent 40% of studies recorded on the register. Our study results are therefore not generalisable to studies not requested by regulatory authorities.

Data from the Register on study status (finalised, ongoing or planned) were collected to allow comparison of studies across time. Information was also collected from the Register on medical condition to be studied (i.e. the disease area of interest listed by the study investigators), data sources used (if applicable), and study scope (effectiveness evaluation, drug utilisation or risk assessment studies). These data were collected from the publicly available information published on the PAS register ( http://www.encepp.eu/encepp/studiesDatabase.jsp).

Studies were classified as either using primary data capture, secondary data capture, or a hybrid approach as used by Engel and colleagues ^7^ who studied PAS protocols and assessments submitted from July 2012 to July 2015 to the EMA Pharmacovigilance Risk Assessment Committee (PRAC). Primary data capture refers to the collection of data specifically for the study. Secondary data is the use of data already collected for another purpose (e.g. administrative or claims databases; medical charts). Studies using a combination of primary and secondary approaches were classed as employing a hybrid approach. Studies using alternative approaches (e.g. interventional studies) to those described were not included, limiting our findings to the aforementioned study designs. It should be noted that study details on the register is not quality checked and therefore misclassification is possible.

Descriptive analysis was performed. Counts and percentages were used for categorical variables.

## Results

As of 18 ^th^ October 2016, a total of 314 studies were identified on the EU PAS register. These studies included 81 (26%) finalised, 160 (51%) ongoing and 73 (23%) planned ( Table 1).

**Table 1.** Type of EMA requested PAS registered in ENCePP by 18 ^th^ October 2016 by study status (finalized, ongoing or planned).

	Risk Assessment		Effectiveness Evaluation		Drug Utilization		Total	
Finalized	42	20%	11	12%	47	35%	81	26%
Ongoing	112	55%	60	64%	59	44%	160	51%
Planned	51	25%	23	24%	27	20%	73	23%
Total	205	100%	94	100%	133	100%	314	100%

### Study scope and design

Of the total studies identified, 205 (65%) included risk assessment in their scope, 133 (42%) included drug utilisation and 94 (30%) included effectiveness evaluation. The same study could cover more than one objective: 6 (2%) studies included drug utilisation and effectiveness evaluation, 36 (11%) drug utilisation and risk assessment, 32 (10%) risk assessment and effectiveness evaluation and 22 (7%) risk assessment, drug utilisation and effectiveness evaluation.

For the risk assessment studies, finalised studies were less common (42; 52% of all 81 finalised studies) than ongoing (112; 70% of all 160 ongoing studies) and planned (51; 70% of all 73 planned studies). For the effectiveness evaluation studies, again finalised studies were not as frequent (11; 14% of all 81 finalised studies) as ongoing (60; 38% of all 160 ongoing studies) or planned (23; 32% of all 73 planned studies). Finally, for the drug utilisation studies, finalised studies were more common (47; 58% of all 81 finalised studies) with 59 (37% of all 160 ongoing studies) ongoing and 27 (37% of all 73 planned studies) planned.

Just over half of the studies (175, 56%) used a primary data capture design, 135 (43%) used a secondary data capture design and 4 (1%) used a hybrid design combining both approaches.

More primary data capture studies were ongoing 102 (64% of all 160 ongoing studies) or planned 49 (67% of all 73 planned studies) than finalised 24 (30% of all of all 81 finalised studies). For secondary data capture, finalised studies were more frequent (57; 70%), as compared to 55 (34%) ongoing and 23 (32%) planned.

Risk assessment and effectiveness studies were more likely to use primary data capture (60% and 85% respectively for primary data capture as compared to 39% and 14% respectively for secondary data capture). The converse was true for drug utilisation studies, where 59% used secondary data capture vs. 39% for primary data capture.

### Secondary data capture studies and data sources used

Of the secondary data capture studies, 117 (87%) used an existing claims or electronic medical record database (the remainder using a chart review approach). 93 (79%) studies used an existing real-world data source based in Europe alone, 9 (8%) studies used European and US data, 14 (12%) studies used US data alone and 1 (1%) study used Canadian data.

A single database was used in 58 (50%) studies, with the remainder using two or more. Where more than one data source was used, this was always from two or more countries. The most frequent established data source used was the United Kingdom’s (UK) Clinical Practice Research Datalink (CPRD, 31%), followed by Nordic (Denmark, Finland, Norway or Sweden) National registries (29%), and The Health Improvement Network (THIN, UK, 18%).

### Disease area and study design

A total of 30 (10%) studies were in the field of type 2 diabetes mellitus, 29 (9%) in cardiovascular disease, 27 (9%) studies in oncology, 6 (2%) in chronic obstructive pulmonary disease (COPD) and 6 (2%) in multiple sclerosis (MS).

For type 2 diabetes mellitus, database studies were more commonly used (80% vs 3% chart review, 3% hybrid and 13% primary data capture). Similar patterns were seen for COPD (83% database vs 17% primary data capture) and cardiovascular disease (59% database vs 38% primary data capture and 3% hybrid design).

For studies in oncology, primary data capture was more likely to be used (85% vs 4% chart review, and 11% database study designs). A similar relationship was seen for MS (83% primary data capture vs 17% database study design).

The full dataset used for analysis, extracted from the PAS register (http://www.encepp.eu/encepp/studiesDatabase.jsp) on the 18th of October 2016 Click here for additional data file. Copyright: © 2017 Carroll R et al. 2017 Data associated with the article are available under the terms of the Creative Commons Zero "No rights reserved" data waiver (CC0 1.0 Public domain dedication).

## Discussion

Our analysis reveals a number of distinct characteristics of studies recorded on the register that can likely be used as a reference for future PAS designs.

Overall there appears to have been an increase in the number of risk assessment studies, as planned and ongoing studies were far more likely to include a risk assessment element than studies that had already been finalised.

Primary data capture study designs were the most commonly implemented study type, and again these were more frequently planned or ongoing, suggesting an increasing use or reflecting the longer nature in general to execute these types of studies. Furthermore, primary data capture were more commonly used for effectiveness and risk assessment studies. The predominance of primary data capture designs is similar to results observed by Engel *et al.,* who studied 189 PAS protocols submitted to the EMA PRAC ^7^.

Secondary data capture was more likely to be used for drug utilisation studies, and this was also described by Engel *et al.* ^7^ Of the secondary studies, the CPRD in the UK was used in nearly a third of all database PAS. Registry data from the Nordic countries was only slightly less commonly used, with the remainder of existing data sources (eg. THIN, in the UK) being less frequently used.

Of the therapeutic areas investigated, type 2 diabetes mellitus, cardiovascular disease and COPD tended to use secondary data capture, whereas for oncology and MS, primary data capture studies were favoured.

Some limiting characteristics of current data sources in Europe are that they may only cover a specific type of patient care (e.g. primary or secondary) and not the full patient pathway. They may not collect data on everything that is needed for PAS objectives, for example in-hospital treatments, clinical endpoints or markers of disease severity. Given PAS studies are often requested on the grounds of exploring sub-groups of patients who may have been treated outside of these settings ^8^, this is a major limitation of some data sources. Indeed, this may explain why primary data capture study designs are becoming more frequent, especially in the field of oncology. Furthermore, that a handful of data sources are very commonly used highlights the limited data landscape in Europe, and the need to improve coverage and access to routinely collected medical data for observational research. On the other hand, given some similarity in data elements captured in currently available datasets, studies using only a single database may have potentially missed a chance to validate any findings or increase statistical power to detect relevant effects. Additionally, some data sources (eg. CPRD) can be linked to other datasets or have an integrated data collection component to provide additional data. Nevertheless, the tendency for effectiveness and risk assessment studies to use primary data capture is also likely a reflection of the fact that this methodology can potentially overcome problems such as confounding, often associated with some secondary designs due to a lack of data availability on potential confounders.

In summary, we show here that using data from the EU PAS Register, study scope and therapeutic area are associated with the choice of study design agreed by health authorities and therapeutic drug makers for the conduct of PAS. While researchers should consider relevant guidelines (ENCePP Guide on Methodological Standards in Pharmacoepidemiology, Guidelines for Good Pharmacoepidemiology Practices of the International Society of Pharmacoepidemiology (ISPE GPP)) when designing PAS, the results of this study can contribute to the planning of future successful PAS.

## Data availability

The data referenced by this article are under copyright with the following copyright statement: Copyright: © 2017 Carroll R et al.

Data associated with the article are available under the terms of the Creative Commons Zero "No rights reserved" data waiver (CC0 1.0 Public domain dedication). 

Dataset 1: The full dataset used for analysis, extracted from the PAS register ( http://www.encepp.eu/encepp/studiesDatabase.jsp) on the 18th of October 2016.

DOI, 10.5256/f1000research.12198.d170864 ^9^
