# Supplementary material for: An analysis of characteristics of post-authorisation studies registered on the ENCePP EU PAS Register
Source: F1000Res. 2017 Nov 10;6:1447. Originally published 2017 Aug 14. [Version 2] doi: 10.12688/f1000research.12198.2 (PMC5698914; doi:10.12688/f1000research.12198.2)
Supplement: Peer review history

# Peer review: Referee response for version 2

**DOI:** 10.5256/f1000research.14284.r27832

**Published:** 2017-11-20

**Authors:** Lex M. Bouter

**Affiliations:** 1 VU University Medical Centre, Department of Epidemiology and Biostatistics, Vrije Universiteit Amsterdam, Amsterdam, Netherlands

My comments and suggestions have been responded to adequately, but the author did not change the manuscript accordingly in all instances.

 Specifically I recommend to add the following study limitations to the discussion section:

- No checks are done for non‐publication, selective publication, and for other discrepancies between protocol and publication for the published studies.
- There is no requirement for studies to have been entered on the register before data collection starts. Similarly later changes to the entry in the register are not identifyable as such.
- The study did not attempt to quantify transparency nor review the methodological rigor of studies, but simply provides an overview of the study designs employed.

I have read this submission. I believe that I have an appropriate level of expertise to confirm that it is of an acceptable scientific standard.

# Peer review: Referee response for version 2

**DOI:** 10.5256/f1000research.14284.r27831

**Published:** 2017-11-13

**Authors:** Xavier Kurz, Thomas Goedecke

**Affiliations:** 1 Pharmacovigilance and Epidemiology Department, European Medicines Agency, London, UK

Most comments have been addressed.

 Minor comments regarding the Introduction:

- "GVP VIII": write in full and provide the link.
- "as a condition for granting marketing authorisation": not only for granting, as PASS can be imposed post-authorisation; write: "as a condition of the marketing authorisation".
- The EU PAS Register is hosted by ENCePP

We have read this submission. We believe that we have an appropriate level of expertise to confirm that it is of an acceptable scientific standard.

# Peer review: Referee response for version 1

**DOI:** 10.5256/f1000research.13205.r26965

**Published:** 2017-10-23

**Authors:** Lex M. Bouter

**Affiliations:** 1 VU University Medical Centre, Department of Epidemiology and Biostatistics, Vrije Universiteit Amsterdam, Amsterdam, Netherlands

The article contains a descriptive analysis of some characteristics of Post-Authorisation Studies registered in the ENCePP EU PAS Register. It’s clearly written and informative within the limited scope the authors set out to study. There are a number of issues that might benefit from further clarification.

 Major issues

- The 314 included studies were on 18 ^th^ October 2016 contained in the ENCePP EU PAS Register ***AND ***concerned a PAS requested by EMA. That makes the reader wonder how many PAS requested by EMA (since a suitable date) were not contained in the Register. And also how many studies contained in the Register were not requested by EMA. In other words: how selective is the sample studied?
- The conclusions come as a surprise because no explicit study questions or hypotheses were formulated. Furthermore, the conclusions are overstated in the sense that both the cross-sectional nature and the lack of a statistical (multivariable) data-analysis make a causal interpretation (‘influence’) unwarranted.
- A serious study limitation is that no checks are done (for the completed studies) for non-publication, and for selective publication and other discrepancies between protocol and publication for the published studies.

Minor issues

- Please explain better what is meant by PAS. Most readers will probably be more familiar with terms like ‘postmarketing surveillance’ and ‘phase IV trials’.
- I assume that entries in the Registry need to be made before the start of data collection. And also that later changes (amendments) to the protocol cannot be made without leaving traces (log file). But this is not clearly explained in the current version of the article.
- The Introduction section suggests that the European pharmacovigilance legislation was introduced to foster transparency and methodological quality of the Post-Authorization Studies. That may be correct, but the current study says very little on transparency and nothing about the methodological quality of PAS.
- The first sentence of the Discussion section suggests that this article will ‘likely be used as reference for future PAS designs’. It completely escapes me why this would be so.

I have read this submission. I believe that I have an appropriate level of expertise to confirm that it is of an acceptable scientific standard, however I have significant reservations, as outlined above.

# Response

**Published:** 2017-11-08

**Authors:** Sreeram Ramagopalan

We thank the reviewers for their comments. We respond specifically to their points below and hope they find our changes acceptable:

Major issues

The 314 included studies were on 18 ^th^ October 2016 contained in the ENCePP EU PAS Register ***AND ***concerned a PAS requested by EMA. That makes the reader wonder how many PAS requested by EMA (since a suitable date) were not contained in the Register. And also how many studies contained in the Register were not requested by EMA. In other words: how selective is the sample studied?

*Response:  Looking at recent figures on the PAS register (06/11/17) 40% (482/1191) fit these criteria. The lack of generalisability of our findings to studies that are not requested by the EMA is now highlighted in the results*

The conclusions come as a surprise because no explicit study questions or hypotheses were formulated. Furthermore, the conclusions are overstated in the sense that both the cross-sectional nature and the lack of a statistical (multivariable) data-analysis make a causal interpretation (‘influence’) unwarranted.

*Response: We had not intended to claim causal inference, but simply describe associations/trends observed. We have removed wording relating to ‘influence’.*

A serious study limitation is that no checks are done (for the completed studies) for non-publication, and for selective publication and other discrepancies between protocol and publication for the published studies.

*Response: The reviewer highlights an important point, but one that is not covered by the aim of our study. We describe the design of studies on the PAS register, we are not addressing the question of whether researchers comply with their study protocols when publishing results (although this would be an interesting analysis!)*

Minor issues

Please explain better what is meant by PAS. Most readers will probably be more familiar with terms like ‘postmarketing surveillance’ and ‘phase IV trials’.

*Response: These terms have been added to the manuscript where the concept of a PAS is first introduced*

I assume that entries in the Registry need to be made before the start of data collection. And also that later changes (amendments) to the protocol cannot be made without leaving traces (log file). But this is not clearly explained in the current version of the article.

*Response: We are not aware of a requirement for studies to have been entered on the register before data collection starts. Amendments can be made but no record of these amendments is made*

The Introduction section suggests that the European pharmacovig)ilance legislation was introduced to foster transparency and methodological quality of the Post-Authorization Studies. That may be correct, but the current study says very little on transparency and nothing about the methodological quality of PAS.

*Response: We agree, our study did not attempt to quantify transparency nor review the methodological rigor of studies, simply provide a overview of the study designs employed.*

**

The first sentence of the Discussion section suggests that this article will ‘likely be used as reference for future PAS designs’. It completely escapes me why this would be so.

*Response: The results presented on study objectives/study design could inform readers on designs most commonly employed to address certain research questions, and therefore potentially inform suitable designs for future studies.*

# Peer review: Referee response for version 1

**DOI:** 10.5256/f1000research.13205.r26352

**Published:** 2017-09-28

**Authors:** Xavier Kurz, Thomas Goedecke

**Affiliations:** 1 Pharmacovigilance and Epidemiology Department, European Medicines Agency, London, UK

The article is well written and provides a descriptive analysis of the EU PAS Register. This type of research is welcome as an evaluation of the usefulness of the EU PAS Register. The article would benefit from a couple of clarifications:

Introduction

- The EU PAS Register is accessible via the ENCePP website which is hosted and maintained by the European Medicines Agency (EMA).
- The term post-authorisation study (PAS) is not exclusive to observational research and may include interventional study designs (e.g. see definition of post-authorisation safety study in GVP VIII), this should be made clear as the introduction refers to historical ‘criticism’ raised mainly in context of non-interventional research. The majority of studies in the EU PAS Register are non-interventional but also a few RCTs  fall under the inclusion criteria applied by the authors for their descriptive study.
- The EU pharmacovigilance legislation and the requirement to publish PAS imposed as a condition of marketing authorisation in a public register came into force in July 2012, not in 2010 (the new legislation was however published in December 2010).
- The authors should explain what they mean by ‘approved PAS’. Regulatory approval of PAS is not a guarantee for successful conduct of a study. The EU pharmacovigilance legislation provides for a regulatory procedure to approve protocols of post-authorisation safety studies (PASS) imposed as a condition of marketing authorisation (EU RMP category 1 and 2 studies) before study start to ensure the objectives of the PASS are met and the protocol follows the format requirements. However, this needs to be distinguished from regulatory supervision of EU RMP category 3 studies where prior protocol approval is not required by law (but may be requested to the marketing authorisation holder voluntarily). The general statement above should be balanced, also in the discussion. Like for any other study, relevant scientific guidance should be considered by marketing authorisation holders and investigators for the development of study protocols, the conduct of studies and the writing of study reports (e.g. ENCePP Guide on Methodological Standards in Pharmacoepidemiology, Guidelines for Good Pharmacoepidemiology Practices of the International Society of Pharmacoepidemiology (ISPE GPP) etc.).

Methods

- Clarify if only studies requested by EMA or also by other EU or non-EU regulators have been included in the descriptive analysis. This is important for the discussion of the results on data sources.
- The authors have chosen 3 distinct study scopes as inclusion criteria, but there might be other studies requested by a regulator which provide insight in trends in study design, data sources and therapeutic areas (a query performed on 27.09.2017 found more than 170 studies requested by a regulator with other scopes). The authors should explain this limitation.

Results

- The presentation of the results on study status, data sources and disease areas may benefit from a breakdown by EU RMP category to show any potential association of with the timelines for conducting studies.
- The authors do not specify how the disease areas were defined (by ATC code?).

Discussion

- The statement that planned and ongoing studies were far more likely to include a safety element is not clear from the presented results; any study in an EU RMP is linked to a safety concern, only with varying scope i.e. effectiveness of measures implemented to manage risks (e.g. educational materials), changes in drug utilisation (e.g. restricted indication) or risk quantification (e.g. frequency of adverse reactions)?
- A limitation of the study that may be worth explaining is that the registration of studies is voluntary (except for imposed PASS), and the entries are not verified as to their accuracy. For examples, it is not checked by ENCePP or EMA whether the RMP category entered is correct and corresponds to the outcome of the regulatory procedure. Therefore, there may be some misclassification. For PASS imposed by regulators, the RMP category field may have been incorrectly populated by the company. 

We have read this submission. We believe that we have an appropriate level of expertise to confirm that it is of an acceptable scientific standard, however we have significant reservations, as outlined above.

# Response

**Published:** 2017-11-08

**Authors:** Sreeram Ramagopalan

We thank the reviewers for their comments. We respond specifically to their points below and hope they find our changes acceptable:

 

- The EU PAS Register is accessible via the ENCePP website which is hosted and maintained by the European Medicines Agency (EMA).

*Response: This has been clarified in the methods.*

- The term post-authorisation study (PAS) is not exclusive to observational research and may include interventional study designs (e.g. see definition of post-authorisation safety study in GVP VIII), this should be made clear as the introduction refers to historical ‘criticism’ raised mainly in context of non-interventional research. The majority of studies in the EU PAS Register are non-interventional but also a few RCTs  fall under the inclusion criteria applied by the authors for their descriptive study.

*Response: This point has been clarified in the introduction*  

- The EU pharmacovigilance legislation and the requirement to publish PAS imposed as a condition of marketing authorisation in a public register came into force in July 2012, not in 2010 (the new legislation was however published in December 2010).

*Response: Text corrected, thank you.*

 

- The authors should explain what they mean by ‘approved PAS’. Regulatory approval of PAS is not a guarantee for successful conduct of a study. The EU pharmacovigilance legislation provides for a regulatory procedure to approve protocols of post-authorisation safety studies (PASS) imposed as a condition of marketing authorisation (EU RMP category 1 and 2 studies) before study start to ensure the objectives of the PASS are met and the protocol follows the format requirements. However, this needs to be distinguished from regulatory supervision of EU RMP category 3 studies where prior protocol approval is not required by law (but may be requested to the marketing authorisation holder voluntarily). The general statement above should be balanced, also in the discussion. Like for any other study, relevant scientific guidance should be considered by marketing authorisation holders and investigators for the development of study protocols, the conduct of studies and the writing of study reports (e.g. ENCePP Guide on Methodological Standards in Pharmacoepidemiology, Guidelines for Good Pharmacoepidemiology Practices of the International Society of Pharmacoepidemiology (ISPE GPP) etc.)

*Response: We have added* *text in* *the discussion to acknowledge this.*

 

- Clarify if only studies requested by EMA or also by other EU or non-EU regulators have been included in the descriptive analysis. This is important for the discussion of the results on data sources.

*Response: This was just EMA only.*


* *

- The authors have chosen 3 distinct study scopes as inclusion criteria, but there might be other studies requested by a regulator which provide insight in trends in study design, data sources and therapeutic areas (a query performed on 27.09.2017 found more than 170 studies requested by a regulator with other scopes). The authors should explain this limitation.

*Reponse* *: This limitation has been noted*

Results

- The presentation of the results on study status, data sources and disease areas may benefit from a breakdown by EU RMP category to show any potential association of with the timelines for conducting studies.

*Response: Unfortunately we did not collect this data in our analysis but agree it would be of interest for future work.*


* *

- The authors do not specify how the disease areas were defined (by ATC code?).

*Response: The disease areas are listed in the EU PAS register. This has been clarified*

Discussion

- The statement that planned and ongoing studies were far more likely to include a safety element is not clear from the presented results; any study in an EU RMP is linked to a safety concern, only with varying scope i.e. effectiveness of measures implemented to manage risks (e.g. educational materials), changes in drug utilisation (e.g. restricted indication) or risk quantification (e.g. frequency of adverse reactions)?

*Response: Risk assessment is meant here (as opposed to drug utilisation, for instance). The text has been updated.*


* *

- A limitation of the study that may be worth explaining is that the registration of studies is voluntary (except for imposed PASS), and the entries are not verified as to their accuracy. For examples, it is not checked by ENCePP or EMA whether the RMP category entered is correct and corresponds to the outcome of the regulatory procedure. Therefore, there may be some misclassification. For PASS imposed by regulators, the RMP category field may have been incorrectly populated by the company. 

*Response: This limitation has been noted*
